# TiO_2_ Self-Assembled, Thin-Walled Nanotube Arrays for Photonic Applications

**DOI:** 10.3390/ma12081332

**Published:** 2019-04-24

**Authors:** Christin David

**Affiliations:** Madrid Institute for Advanced Studies in Nanoscience (IMDEA Nanoscience), C/Faraday 9, 28049 Madrid, Spain; christin.david@imdea.org

**Keywords:** TiO_2_ nanotubes, photonic crystals, optical engineering, theory and simulation

## Abstract

Two-dimensional arrays of hollow nanotubes made of TiO${}_{2}$ are a promising platform for sensing, spectroscopy and light harvesting applications. Their straightforward fabrication via electrochemical anodization, growing nanotube pillars of finite length from a Ti foil, allows precise tailoring of geometry and, thus, material properties. We theoretically investigate these photonic crystal structures with respect to reduction of front surface reflection, achievable field enhancement, and photonic bands. Employing the Rigorous Coupled Wave Analysis (RCWA), we study the optical response of photonic crystals made of thin-walled nanotubes relative to their bare Ti foil substrate, including under additional charge carrier doping which might occur during the growth process.

## 1. Introduction

Titanium dioxide (TiO${}_{2}$) nanostructures and their electrical, chemical and optical properties are of high interest in various scientific fields [1]. The electrochemical growth of TiO${}_{2}$ nanotubes from a Ti foil allows studying self-assembled nanotube (NT) arrays with a well-defined geometrical configuration depending on growth parameters applied during the anodization process. Hence, thick and thin-walled, ordered and disordered arrays, as well as advanced geometries with increased nanoscopic surface roughness such as bamboo or double-walled nanotubes have been achieved [2]. Annealed crystalline TiO${}_{2}$ NTs show an increased charge carrier doping level allowing to further improve electron transfer efficiency for bio-electrochemistry applications [3]. It was demonstrated that additional, free electrons can yield high optical field enhancement in densely packed, thin-walled TiO${}_{2}$ NT arrays, independent from the chemical environment [4].

**Figure 1:**
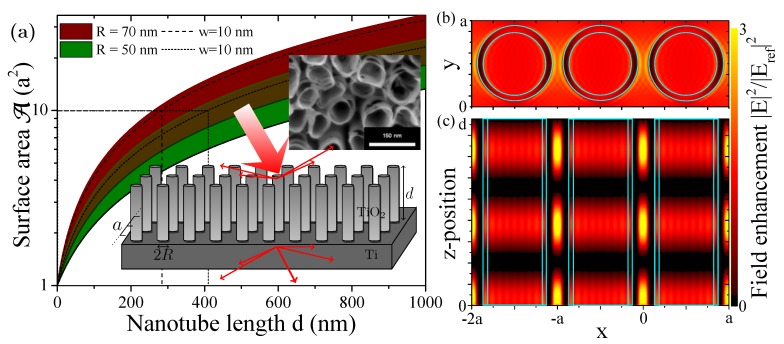
(**a**) Increase in surface area as a function of nanotube length *d* for two different outer radii *R* and lattice period a=160nm. The shaded areas span the minimum to maximum achievable surface area from solid nanopillars (wall thickness w=R) to ultrathin walls w→0, the dashed lines mark w=10nm. Insets: SEM image of real geometry after self-assembly of hollow TiO${}_{2}$ nanotubes on a Ti substrate [4] and illustration of the idealized structure used in our modeling; (**b**,**c**) Field enhancement of a d=500nm NT with R=70nm for three neighboring unit cells (**b**) at the top of the NTs z=d and (**c**) in the orthogonal plane in the center of the unit cell at y=a/2.

Such arrays have wide applications in sensing [5], as filters and nanosized test tubes in biomedicine [6], as photonic crystal fiber lasers and demultiplexers [7,8,9], and as nanostructured electrodes for (surface enhanced Raman) spectroscopy [3,4]. In addition, the possible enhancement of reaction or transport rates due to a large surface area, see Figure 1a, strong electron confinement and short diffusion paths make them highly interesting nanostructures for (photo-)catalysis [10,11,12,13] and photovoltaics (PV) [14,15,16,17,18]. While typically grown on Ti substrates or alloys, TiO${}_{2}$ NTs can be produced as membranes through a lift-off process [2]. This technique allows employing them on different substrates such as Si or GaAs for solar cells. A further advantage for use in bionanotechnology, catalysis and related fields is the biocompatiblity of the material [3,4], which makes an additional protective coating of the obtained oxide unnecessary. Self-assembled nanostructures are favorable with view to their fabrication costs in particular in a highly competitive industry such as PV technology [19,20]. The high refractive index (RI) of TiO${}_{2}$ makes this an interesting material for a broad range of further photonic and hybrid applications, i.e., exploiting plasmon-assisted enhancement effects by combining with metal nanoparticles or planar waveguide structures [21,22]. These setups are typically used to enhance intrinsically low quantum efficiencies, e.g., in rare earth transition rates [18], and to sensitize the UV-active TiO${}_{2}$ towards visible light [11]. The nanotubular geometry adds directionality to incoming light improving charge transfer towards an electrode or photon scattering towards a photo-active substrate [16,17]. The sensitivity in such photonic crystal structures is high and robust with respect to fabrication defects due to collective optical modes in the regular lattice structure [23].

The NT arrays obtained can show a high degree of order and regularity which allows studying and optimizing their properties with available modeling tools. This article investigates systematically the optical modes of such structures for a wide parameter range. We take a detailed look at the optical properties of photonic crystals made from regular TiO${}_{2}$ NT arrays with an emphasis on the influence of the NT wall thickness and charge carrier doping during the anodization [4,13], two parameters not typically available in nanostructures fabricated with, e.g., lithographic techniques. The reflection and near-field spectra are obtained from Rigorous Coupled Wave Analysis (RCWA) [24,25,26] simulations. The RCWA method is capable of describing the electro-optical properties of regular nanostructures in a multilayered device including inhomogeneous unit cells [27] and nonclassical, mesoscopic electron dynamics [28,29]. In a recent collaboration, we demonstrated that this method is highly apt to explain optical properties and field enhancement in experimental findings in hollow nanotube arrays [4]. We lay out the theoretical aspects of the presented simulations in the next section and discuss our findings in detail afterwards.

## 2. Methods

### 2.1. System under Investigation

**Figure 2:**
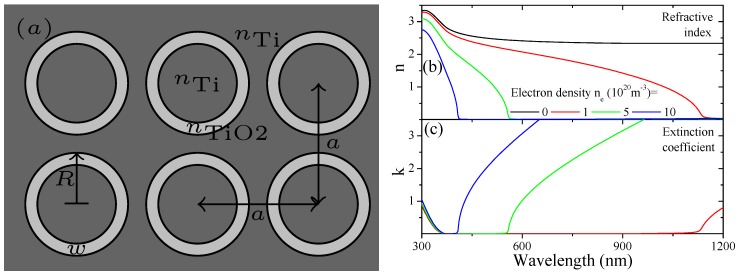
(**a**) Top view of the simulated, two-dimensional structure depicting the lattice period *a*, outer radius *R* and wall thickness *w*; (**b**) Refractive index *n* and (**c**) extinction coefficient *k* of TiO${}_{2}$ for different levels of electron doping and the original data.

Two-dimensional arrays of hollow TiO${}_{2}$ nanotubes on a large Ti substrate are considered, as depicted in the insets in Figure 1a. Our simulations allow us to study the electromagnetic field around these structures, see Figure 1b,c for a top and side view. Figure 2a shows in more detail the square array geometry. The lattice period *a* defines the distance between nanotube pillars. The TiO${}_{2}$ nanotubes have an outer radius of *R* and their wall thickness and height are *w* and *d*, respectively.

The calculations were performed using frequency-dependent, tabulated RI data in the visible spectrum for TiO${}_{2}$ from Ref. [30] measured from thin films and Ti from Ref. [31] from bulk material. In order to obtain smooth data points, interpolation between available measured data was used. The refractive index *n* and related extinction coefficient *k* for TiO${}_{2}$ are shown in Figure 2b,c, respectively. Within the visible spectrum, the RI is n=2.33±0.31 with vanishing *k* for TiO${}_{2}$ and 2.02+i2.79±(0.83+i0.98) for the large Ti substrate.

Experimentally [4,13] it was observed that additional charge carriers are injected into the system during the growth process of TiO${}_{2}$ nanotubes. We can account for this effect considering RI data modified by the presence of free electrons in a Drude model (in Gauss units)

(1) $$\begin{array}{c} \epsilon \left(\omega \right)=\epsilon_{{\mathrm{TiO}}_{2}}-\frac{4\pi n_{e}}{\omega (\omega +i\gamma )}. \end{array}$$

We assume a small, intrinsic damping γ=0.01eV, a varying carrier density of $n_{e}$. This behavior explains recent experimental findings [4] and allows identifying the doping level and conductivity of fabricated samples. The original material data and modification due to varying the free electron density are shown in Figure 2b,c.

The overall parameter space investigated is summarized in Table 1.

### 2.2. Rigorous Coupled Wave Analysis for Hollow Nanotubes

We use the RCWA [24,25,26,27,28,29] to calculate the optical properties of periodic two-dimensional TiO${}_{2}$ nanotube square arrays. First, infinite NT arrays made from a geometry defined via its unit cell are described within this plane-wave expansion approach. Finite arrays are obtained applying the usual boundary conditions for an electromagnetic field defining the length of the arrays. Calculations on multilayers, in particular through the inclusion of a substrate, are performed with the scattering matrix method. Thus, we arrive at a realistic description of regular nanotube arrays as depicted in the inset of Figure 1a and in its top view in Figure 2a.

The main ingredient is the Fourier transform of the two-dimensional unit cell, formally decomposing its spatial permittivity $\epsilon (\omega ,\vec{R})$ into plane waves in reciprocal space

(2) $$\begin{array}{c} \epsilon \left(\omega ,\vec{R}\right)=\sum_{\vec{G}}\epsilon_{\vec{G}}e^{i\vec{G}\vec{R}}. \end{array}$$

We derive the Fourier coefficients from

(3) $$\begin{array}{c} \epsilon_{\vec{G}{\vec{G}}^{\prime }}=\frac{1}{a^{2}}\int_{0}^{a}dx\int_{0}^{a}dy\;\epsilon \left(\omega ,\vec{R}\right)e^{-i\left(\vec{G}-{\vec{G}}^{\prime }\right)\left(\vec{R}-{\vec{R}}_{0}\right)}, \end{array}$$

where we already assumed a square lattice and reciprocal wave vectors $\vec{G}=2\pi \left(n\hat{x}+m\hat{y}\right)/a$ with $n,m\in Z_{0}$ for a lattice period of *a* and circular particles at a position ${\vec{R}}_{0}=\left(x_{0},y_{0}\right)$ within the unit cell. It is convenient to choose the center of the unit cell here, i.e., $x_{0}=y_{0}=a/2.$

The Fourier transform is analytical for spherical shapes (and indeed also for elliptical particles via coordinate transformations, see [32]) and can account for (i) unit cells comprised of inhomogeneous particle distributions [27] and (ii) core-shell structures with *N* interfaces

(4) $$\begin{array}{c} \epsilon \left(\omega ,\vec{R}\right)=\epsilon_{0}+\sum_{n=1}^{N}\left(\epsilon_{n}-\epsilon_{n-1}\right)\Theta (R_{n}-|\vec{R}-{\vec{R}}_{0}\left|\right). \end{array}$$

This can be solved in complete analogy to the case of a single solid particle disk [27,32], such that

(5) $$\begin{array}{cc} \epsilon_{\vec{G}{\vec{G}}^{\prime }} & =\delta_{\vec{G}{\vec{G}}^{\prime }}\left[\epsilon_{0}+\sum_{n=1}^{N}\frac{\pi R_{n}^{2}}{a^{2}}\left(\epsilon_{n}-\epsilon_{n-1}\right)\right]+\sum_{n=1}^{N}\frac{2\pi R_{n}^{2}}{a^{2}}\left(\epsilon_{n}-\epsilon_{n-1}\right)e^{i\left(\vec{G}-{\vec{G}}^{\prime }\right)R_{0}}\frac{J_{1}\left(\right|\vec{G}-{\vec{G}}^{\prime }\left|R_{n}\right)}{|\vec{G}-{\vec{G}}^{\prime }|R_{n}}, \end{array}$$

with Bessel functions $J_{n}\left(x\right)$ of *n*’th order and $\int d\phi e^{-i|\vec{G}-{\vec{G}}^{\prime }|R\cos\phi }=2\pi J_{0}\left(\right|\vec{G}-{\vec{G}}^{\prime }\left|R\right)$. The first term defines diagonal ($\vec{G}\equiv {\vec{G}}^{\prime }$), the second term off-diagonal entries. These results remain analytical and the computational effort is only increased by the summation. Arrays of thin-walled nanotubes as discussed here have N=2 interfaces defined by an outer $R_{1}\equiv R$ and an inner radius $R_{2}\equiv R-w$, where *w* is the wall thickness of the nanotube. The filling equals the ambient material $\epsilon_{2}=\epsilon_{0}=1$ (air/vacuum) throughout this article for convenience. With the nanotubes described as above, we follow the standard RCWA and scattering matrix procedure [24,25,26]. Convergence is reached quickly in this dielectric system. Plane-waves of 169 (n,m∈{−6…6}) are usually enough to produce converged far-field spectra, 529 (n,m∈{−11…11}) was used for near-field data, but convergence was checked for up to 961 (n,m∈{−15…15}) plane-waves, in particular, where plasmonic properties emerge when free carrier doping was assumed.

The numerical simulations were realized with in-house C/C++ codes and run on a local high-performance computation cluster.

### 2.3. Field Enhancement Factor (EF)

We normalize the electric field to the incoming light field and with respect to the field obtained for a flat Ti electrode ${\vec{E}}_{\mathrm{ref}}$ (d=0 case). The evaluation is done either

(i) directly as $\mathrm{EF}={\left|\vec{E}\left(x,y,z\right)\right|}^{2}/{\left|{\vec{E}}_{\mathrm{ref}}\right|}^{2}$ used in field maps, or
(ii) as 2D averaged EF, taking the mean over the unit cell area (x-y-plane)
  (6) $$\begin{array}{c} {\mathrm{EF}}^{\mathrm{av},2D}\left(z\right)=\frac{1}{a^{2}{\left|{\vec{E}}_{\mathrm{ref}}\right|}^{2}}\int_{0}^{a}dx\int_{0}^{a}dy{\left|\vec{E}\left(x,y,z\right)\right|}^{2}, \end{array}$$

typically, at the top of the NTs (z=d). If not stated otherwise, an interparticle distance a=160nm, a particle diameter of 2R=140nm and a wall thickness of w=10nm are used. This defines a dense NT array where the TiO${}_{2}$ pillars are not yet touching.

## 3. Results and Discussion

**Figure 3:**
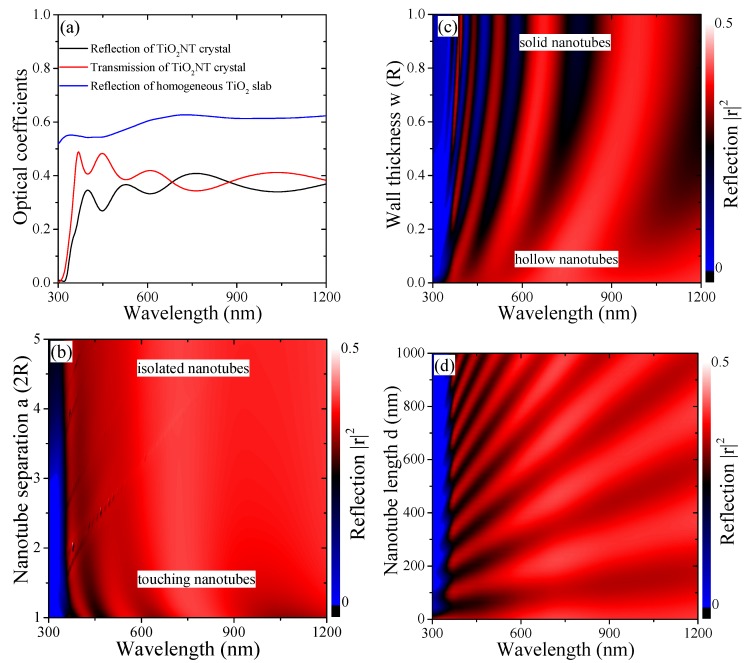
(**a**) Optical spectra of a TiO${}_{2}$ nanotube pillar array with d=500nm, a=160nm, R=70nm and w=10nm comparing to the reflection of a homogeneous TiO${}_{2}$ slab of the same thickness. Studying the reduction in surface reflection for spectra of TiO${}_{2}$ NT arrays as a function of (**b**) nanotube wall thickness $w\in \{0^{+}\ldots R\}$, (**c**) lattice parameter a∈{2R…10R}, and (**d**) nanotube length d∈{0…1000} nm.

The hollow TiO${}_{2}$ nanotube structures discussed in this article are shown in an SEM (Scanning Electron Microscopy) image [4] and the related simulated structure is likewise depicted in the inset of Figure 1a. We consider the active surface A of a unit cell with and without nanotubes in Figure 1a. For nanotubes with outer radius *R* and wall thickness *w*, the surface area becomes $A=a^{2}+d2\pi \left(2R-w\right)$. Hence, the active surface increases linearly with the tube length from $a^{2}$ for the flat device while the slope flattens with increasing unit cell size. A ten-fold surface area is reached at nanotubes of a few hundred nanometers long which is well within experimentally achievable setups [2,4]. Figure 1b,c show the electromagnetic field enhancement along the xy- and xz-planes, respectively, revealing an oscillatory structure for field maxima in between the nanotubes and inside the hollow structures along the nanotube length. Typically, the field inside the NT wall is supressed and an enhancement is observed directly at the TiO${}_{2}$ surface. The overall enhancement is small compared to other material choices, but stable for the entire UV and VIS spectrum. It can be increased through doping of the structure, which we discuss later on. This and the straightforward fabrication and geometrical tuning of TiO${}_{2}$ nanotubes makes this an ideal material system to study photonic crystals made of hollow nanotube arrays. One important property of photonic crystals as front layers for light harvesting devices is their ability to reduce surface reflection, similar to e.g., “Black Silicon” [33,34]. This is accompanied by an increase in the efficiency of forward scattering towards the photoactive layer typically lying underneath the nanostructured front surface. This is investigated in Figure 3 over the VIS spectrum for different geometrical parameters. In Figure 3a, the reflection and transmission of a specific configuration (details in the figure caption) is compared to the reflection of a solid TiO${}_{2}$ slab of the same thickness. There is a reduction in the reflection by about 20% absolute. Due to the macroscopic crystal structure, transmission bands form and tuning the geometrical parameters can suppress the surface reflection further. In Figure 3b, we study the reflection spectra as a function of the nanotube wall thickness *w*. Low reflection is achieved below λ<600nm and more pronounced for a larger wall thickness. This observation makes the wall thickness an intriguing parameter, not typically accounted for in photonic crystal structures due to the use of solid nanoparticles. At the same time, stronger optical lattice effects are observed for small NT separations, i.e., lattice parameter *a*, see Figure 3c, where the smallest value possible is a≡2R when NT surfaces touch. Furthermore, Figure 3d shows this dependence for the NT length *d*. The reflection minima are hereby linearly shifting with the wavelength, rendering an optimization somewhat impractical. Shorter NT lengths are most promising, since the shifted optimum is varying slowly across the spectrum. This oscillation with the nanotube length in the spectrum is of similar origin to the observations of spatial oscillations of the electromagnetic field in Figure 1c, which we discuss next in more detail.

**Figure 4:**
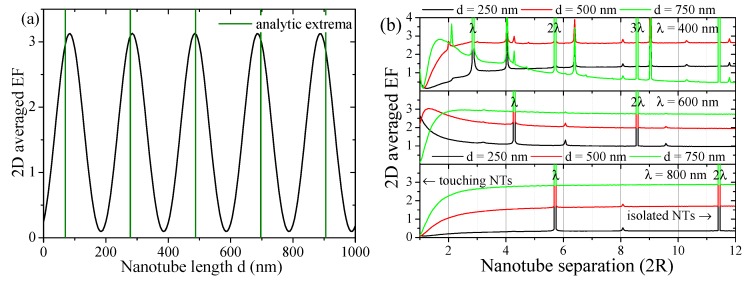
(**a**) Averaged field enhancement $E^{\mathrm{av},2D}$ in the unit cell at the top of the NTs z=d at λ=600nm as a function of nanotube length compared to analytic maxima, Equation (7); (**b**) Averaged enhancement factor $|{\vec{E}}^{\mathrm{av},2D}{|}^{2}/{\left|{\vec{E}}_{\mathrm{ref}}\right|}^{2}$ within the unit cell as a function of the nanotube lattice parameter a∈{2R…24R} with R=70nm for several nanotube lengths *d* and excitation wavelengths λ. Divergences are observed where the ratio a/R is an integer and, in particular, where the lattice parameter coincides with a multiple of the wavelength.

The maximum value of the intensity within the unit cell (evaluated at the front surface (z=d)) follows a sinusoidal law, shown in Figure 4a, related to Bragg diffraction

(7) $$\begin{array}{c} |\vec{E}{|}^{2}∼|{\vec{E}}_{0}{|}^{2}\sin^{2}\left(kd\right)={\left|{\vec{E}}_{0}\right|}^{2}\sin^{2}\left(\sqrt{\epsilon_{\vec{G}\vec{G}}}\frac{2\pi }{\lambda_{\mathrm{exc}}}d\right). \end{array}$$

**Figure 5:**
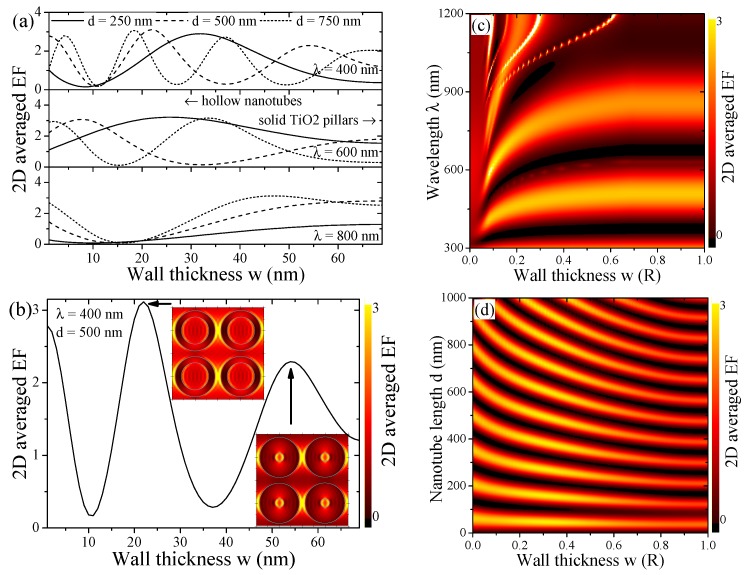
As a function of the wall thickness *w*, the averaged field enhancement $|{\vec{E}}^{\mathrm{av},2D}{|}^{2}/{\left|{\vec{E}}_{\mathrm{ref}}\right|}^{2}$ is presented for (**a**) several NT lengths *d* and excitation wavelengths λ with a detailed look at a specific configuration in (**b**), adding the field maps for two of the maxima; (**c**) Similar to Figure 3b, we show its full spectral dependence as a contour; (**d**) The full contour as a function of the NT length *d*, showing the maxima of Figure 4a shifting in a nonlinear manner with decreasing wall thickness *w*.

The field enhancement in the xy-plane of the nanotube array shows a general dipole field distribution around the nanotubes, see Figure 1b and insets in Figure 5c. Hereby, the roots are characterized by $d=n\lambda_{\mathrm{exc}}/\left(2\sqrt{\epsilon_{\vec{G}\vec{G}}}\right)$ with integer *n*. Hence, the maxima are found for $n\pm \frac{1}{2}$ values. The position of these analytic maxima as a function of the nanotube length are marked in Figure 4a. Discrepancies can be expected, since taking the diagonal value $\epsilon_{\vec{G}\vec{G}}$ of the permittivity matrix, Equation (5) in the analytic approximation becomes less valid for longer nanotubes where higher order Fourier components become increasingly important, see the small descrepancies in Figure 4a.

Further analytically comprehensive traits of this crystal structure are shown in Figure 4b. The 2D averaged field enhancement is shown as a function of the NT separation for a number of wavelengths and NT lengths. Here, we observe a number of divergent resonances, which can be understood in an empty lattice picture. Resonant bands are found where $|{\vec{k}}_{\|}-\vec{G}|=k$. However, the parallel momentum ${\vec{k}}_{\|}=0$, so that the reciprocal lattice vector $|\vec{G}|=2\pi n\;/\;a$ equals the wave vector k=2π/a, leading to the condition λn=a. Hence, where the lattice parameter *a* hits a multiple of the wavelength λ, we observe strong resonant field enhancement promoted by geometrical lattice conditions. Weaker, non-divergent resonances are often observed close to the condition of a=2nR at lower wavelengths, i.e., higher photonic energies. Here, the lattice parameter is a multiple of the NT diameter. However, since we study hollow NTs and not solid nanopillars, these resonances are less pronounced.

Figure 5 studies in depth the dependence of the TiO${}_{2}$ thin-walled nanotube arrays on the wall thickness, a degree of freedom not typically available in nanoarrays. Figure 5a selects a few wavelengths and nanotube lengths, comparing the 2D averaged field enhancement. For each such pairing, one or several maxima and minima can be found, completely suppressing the field enhancement in this particular 2D-plane. This behavior is shifting when moving the z-position, as seen in Figure 1c. We take a closer look at one of these pairings in Figure 5b, adding the near-field maps of the two observed maxima and indicating the wall thickness. Photonic bands are evident in Figure 5c, stabilizing quickly for a wall thickness above 0.2R at the lower wavelength band and above 0.4R at the higher wavelength band, however, showing largest field enhancement for low wall thickness. Note that for sufficiently thin walls, optical surface modes from both sides can couple and lead to higher local fields. Larger wavelengths only maintain high field bands for low wall thickness. The Bragg law derived from Equation (7) and discussed above for Figure 4a is modified with the wall thickness, which we show in Figure 5d. The distance of the maxima with respect to the nanotube length becomes larger for decreasing wall thickness while increasing in intensity.

**Figure 6:**
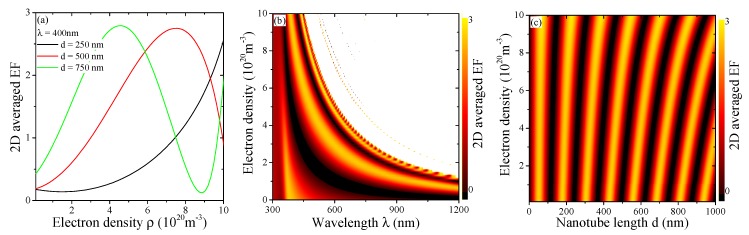
(**a**) Averaged enhancement factor as a function of the doping level of the system for a=160nm for several nanotube lengths and λ=400nm; (**b**) Contour presenting the data from (**a**) for full spectra; (**c**) Contour presenting the data from (**a**) as a function of nanotube length, cp. Figure 4a.

Due to the additional electron doping that occurs during the growth process [4,13], we consider RI data modified by the presence of free electrons in a Drude model. This reduces the permittivity and thus the sine in Equation (7) is shifted. Figure 6 explores the dependence of the optical properties of TiO${}_{2}$ nanotubes on the doping level. Figure 6a demonstrates that doping increases the 2D averaged field enhancement initially, but can drop afterwards. We show full spectra in Figure 6b where a high field enhancement plateau is revealed that is connected to the transition of the permittivity from a dielectric to a metal one, i.e., increasingly negative values in Equation (1). For larger wavelengths this is achieved at already low doping levels, but clearly, additional charge carriers are needed to achieve this transition. In Figure 6c, we look again at the Bragg law derived from Equation (7). Here, we observe a bending and shifting of the maxima of the sine function of Figure 4a similar to Figure 5d, this time with the doping level. As before, this is tightly connected to the fact, that the effective permittivity of the TiO${}_{2}$ nanotube crystal, Equation (5), is modified through either geometry (wall thickness in Figure 5d) or carrier density.

## 4. Conclusions

In summary, we have studied optical properties of hollow TiO${}_{2}$ nanotube arrays, whose fabrication has become a standard tool. A finite wall thickness adds another degree of freedom in the design of such structures as compared to typically used solid nanopillar arrays. In addition, we account for a low doping level ocurring during the growth process from a Ti foil, which was previously used to explain experimental observations. Both these aspects modify the effective permittivity of the setup and can thus be used to maximize local field enhancement at specific wavelengths. Higher doping levels yield plasmon-like behavior, yielding increasingly high local fields, and make these structures interesting for biocompatible sensing, photovoltaics, catalysis and spectroscopy applications, such as surface enhanced Raman spectroscopy [4]. Where high losses in standard metal plasmonic materials limit their application, in particular in light harvesting applications, alternative materials with low intrinsic losses enable different routes. While solid TiO${}_{2}$ is active in the UV spectrum, nanostructuring, as demonstrated in this article with hollow nanotube arrays, allows bringing interesting optical properties into the visible window. However, it should also be noted that the doping achieved naturally during the growth process is too low, to alter material properties significantly towards plasmonics. Next to dioxides like TiO${}_{2}$, other mentionable materials with similar opportunities include conductive nitrides [35,36,37].

We demonstrated that TiO${}_{2}$ NT arrays act as photonic crystals forming specific photonic bands. They are able to improve forward scattering, i.e., reduce front surface reflection, and together with the low cost self-assembly, they make an attractive alternative for photovoltaics and photocatalysis supported by nanostructures.

## Figures and Tables

**Table 1:** Range of different geometrical parameters and illumination conditions under study.

	Wavelength λ	Radius *R*	Pitch *a*	Wall Thickness *w*	Height *d*	Electron Density $n_{e}$
min	300 nm	50 nm	2*R*	0 nm	0 nm	0 m${}^{-3}$
max	1200 nm	70 nm	24*R*	R	1000 nm	10${}^{21}$ m${}^{-3}$
